# Recent Progress in Thin-Film Transistors toward Digital, Analog, and Functional Circuits

**DOI:** 10.3390/mi13122258

**Published:** 2022-12-19

**Authors:** Seongjae Kim, Hocheon Yoo

**Affiliations:** Department of Electronic Engineering, Gachon University, Seongnam 13120, Republic of Korea

**Keywords:** organic semiconductors, metal oxides, gate dielectrics, thin-film transistors, flexible circuits, plastic substrates

## Abstract

Thin-film transistors have been extensively developed due to their process merit: high compatibility with various substrates, large-area processes, and low-cost processes. Despite these advantages, most efforts for thin-film transistors still remain at the level of unit devices, so the circuit level for practical use needs to be further developed. In this regard, this review revisits digital and analog thin-film circuits using carbon nanotubes (CNTs), organic electrochemical transistors (OECTs), organic semiconductors, metal oxides, and two-dimensional materials. This review also discusses how to integrate thin-film circuits at the unit device level and some key issues such as metal routing and interconnection. Challenges and opportunities are also discussed to pave the way for developing thin-film circuits and their practical applications.

## 1. Introduction

Tremendous efforts have been made to investigate and develop thin-film transistors (TFTs) using various thin-film materials, including small molecules [1,2,3], polymers [4,5], metal oxides [6,7], transition metal dichalcogenides (TMDs) [8,9], graphene [10,11], carbon nanotubes (CNTs) [12,13] and perovskite materials [14,15]. With respect to the manufacturing perspective, the advantage of TFTs compared to conventional silicon-based metal-oxide field-effect transistors (MOSFETs) is that TFTs can be deposited on various substrates, including transparent glasses [16,17] and flexible plastics [18,19,20]. Such excellent compatibility with these substrates allows them to be used in many applications: biosensors [21,22], healthcare [23,24], building crack monitoring [25], e-skin [26], and e-nose [27].

Nevertheless, compared to conventional silicon MOSFETs, the low charge mobility and difficulty in scaling limit their applications. For this reason, a strategy that maximizes the advantages of TFT is needed rather than completely replacing the conventional silicon MOSFET with TFT. For example, in terms of flexible, transparent properties and cost, TFTs can outperform silicon MOSFETs because TFTs have superior substrate compatibility [28,29,30] and enable low-cost processes [31,32]. In addition, TFTs are also available for large-area processes [33,34] and various sensor applications [35,36,37,38]. On the other hand, there is a technological requirement that must be satisfied for practical application development using TFTs: the development of complex circuits, such as digital and analog circuits, based on uniformity in large-area processes beyond unit-level demonstrations. Although it is possible to investigate whether a specific material has potential as a TFT material by implementing a unit-device level, but there are still questions about the extension to a practical system. Accordingly, not only the transistor operation of each TFT but also implementations at the circuit level, composed of several TFTs, are required.

In this context, this review provides an overview of recent efforts in thin-film circuits. Results for the latest thin-film circuit implementation are highlighted. Digital and analog thin-film circuits using CNTs, organic electrochemical transistors (OECTs), organic semiconductors, metal oxides, and TMDs are revisited. Furthermore, this review discusses how to integrate thin-film circuits on a unit-device level and some key issues, such as metal routing and interconnections. Challenges and opportunities are also suggested to pave the way for developing thin-film circuits and their practical applications.

## 2. Digital Thin-Film Circuits

### 2.1. CNT-Based Thin-Film Digital Circuits

**Figure 1:**
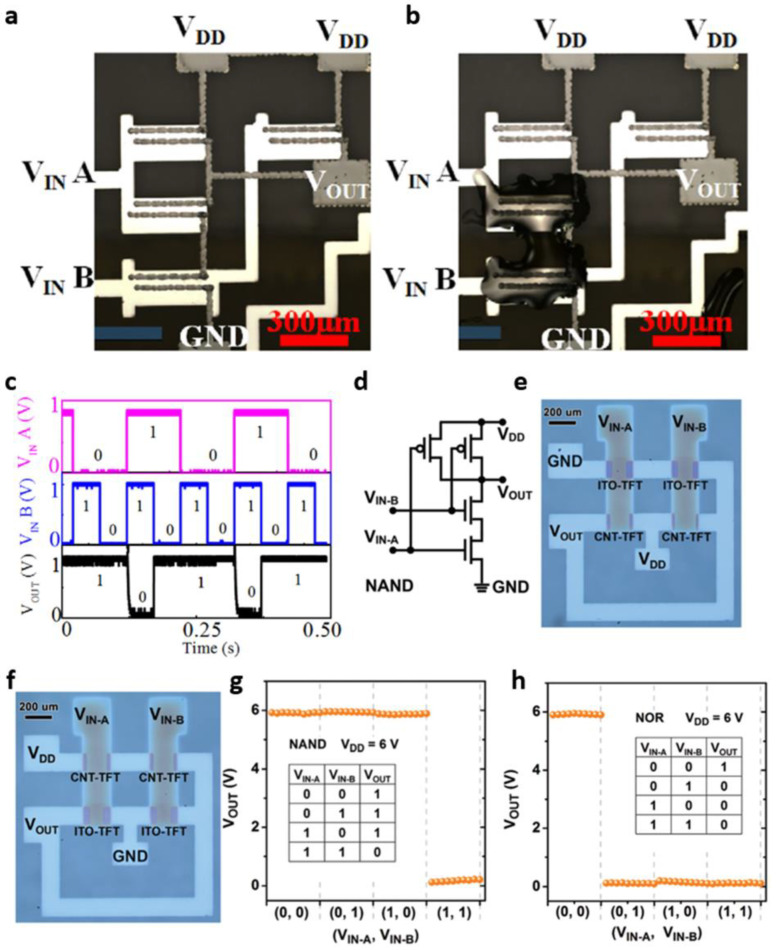
CNT-based digital thin-film circuits. CNT-based NAND circuit (**a**) before and (**b**) after drop-casting of epoxy amine ink and (**c**) output of NAND gate for two final input voltages (A, B). Various SAMs on the SiO_2_ surface (adapted from [42] with permission from Elsevier). (**d**) NAND circuit diagram based on ITO and CNT TFTs. Optical microscopy images of (**e**) NAND and (**f**) NOR circuits based on ITO and CNT TFTs, and (**g**,**h**) logic input/output characteristics of each circuit (adapted from [43] with permission from RSC Publishing).

Single-walled carbon nanotubes (SWCNTs) have an energy bandgap of ~2 eV, which is typically used as a semiconductor film [39,40]. Many efforts have been made to develop CNT-based TFTs owing to their transparency and easy-to-fabricate process with solution processability [40,41]. In this regard, progress in implementing CNTs-based circuits has been reported. This section summarizes recently reported inverter, NAND, or NOR logic gate results using CNTs. As the first example, Wei et al. in 2020 presented complementary *p*-type and *n*-type CNT transistors and their NAND and NOR digital circuit blocks [42]. They selectively deposited CNT networked layers on the targeted channel region by inkjet printing. The CNT transistor was changed from *p*-type operation to *n*-type operation when an epoxy amine ink, as the chemical dopant, encapsulated the CNTs. Using this approach, *p*-type or *n*-type CNT transistors can be made depending on the existence of epoxy amine encapsulation. A logic inverter based on the described above complementary CNT-based transistors was demonstrated. When *V*_DD_ = 1 V, a DC gain of >20 V/V was obtained, and the high noise margin (NM_H_) and low noise margin (NM_L_) were 0.4 V and 0.42 V, respectively. NAND gates were also implemented by constructing two *p*-type CNT-based transistors and the epoxy amine ink-encapsulated *n*-type CNT-based transistors (Figure 1a,b). The characteristics of the fabricated complementary CNT-based NAND circuit are shown in Figure 1c, in which a NAND gate that outputs logic ‘0′ only when both input voltages are logic ‘1′ was successfully demonstrated. In addition, the implemented complementary CNT-based logic circuit maintained its initial characteristics even after three months.

As another approach to attaining complementary circuits, a hybrid thin-film circuit using *p*-type CNT-based transistors and *n*-type indium–tin–oxide (ITO)-based transistors was attempted [43]. Zou et al. reported a complementary logic circuit by combining CNTs and ITO. After CNT transfer, ITO was deposited by sputtering, which completed the complementary integration with the CNTs and ITO as *p*-type and *n*-type transistors [43]. They constructed NAND and NOR circuits using the two *p*-type CNT-based transistors and another two *n*-type ITO-based transistors (Figure 1d–h). The constructed NAND and NOR exhibited proper output values depending on the input value combination of *V*_A_ and *V*_B_. In addition, the fabricated complementary CNT and ITO hybrid inverter was controlled by UV light signal. Depending on the UV light exposure, the inverter circuit determined its proper logic operation or 0 V-only value of the output, highlighting the feasibility of optoelectronic logic circuit operation. In the conventional method, two *p*-type and two *n*-type TFTs are required to implement a circuit for NAND operation. However, the proposed method suggests that NAND operation is possible in a typical inverter circuit if the photoresponse characteristics of the TFT are utilized.

### 2.2. OECT-Based Thin-Film Digital Circuits

**Figure 2:**
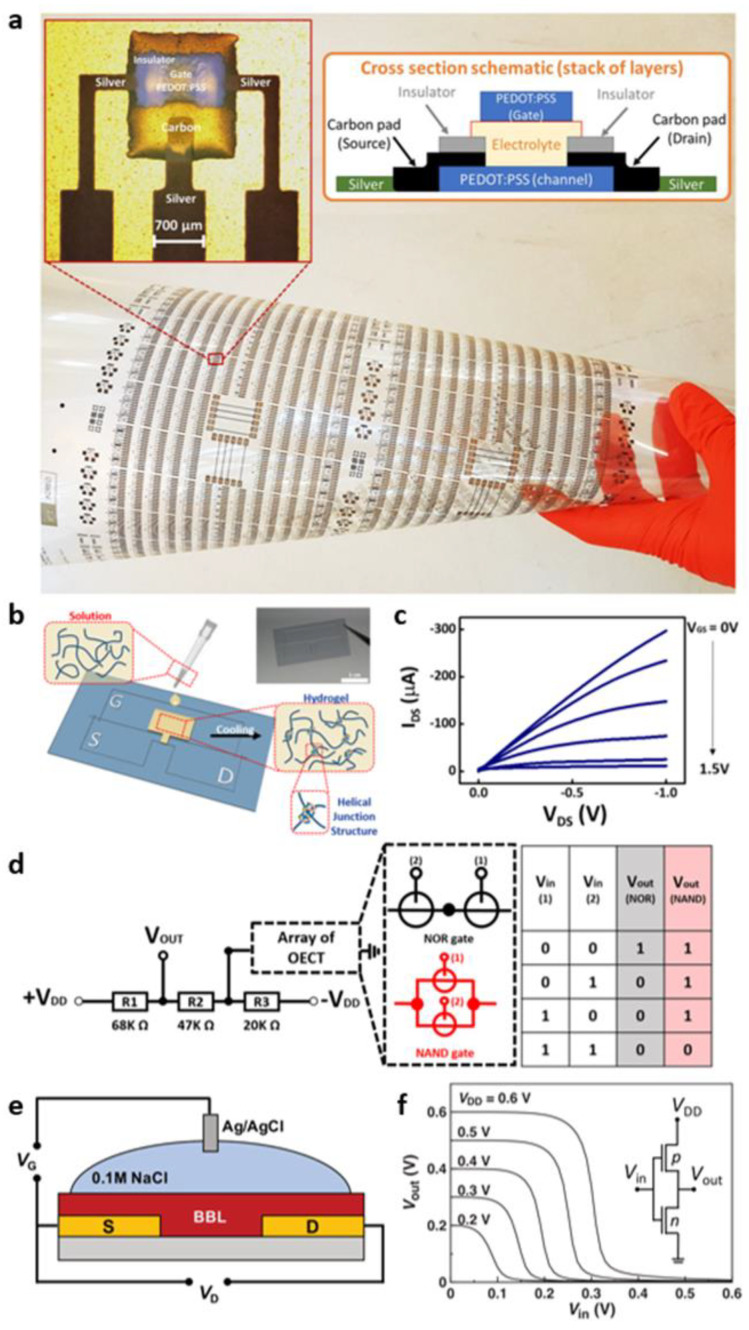
OECT-based digital thin-film circuits. (**a**) An optical microscopy image of 760 OECTs and a single device fabricated on a flexible substrate, and an introduction to each layer (adapted from [44] with permission from Springer Nature). (**b**) Schematic and optical image of gelatin electrolyte-based OECT and (**c**) its output curve. (**d**) Schematic and truth table of NOR and NAND gates in which two gelatin-OECTs were configured in series or parallel structure (adapted from [45] with permission from the American Chemical Society). (**e**) Device schematic diagram of BBL-based *n*-type OECT. (**f**) Input/output characteristics of complementary inverters implemented with BBL-based *n*-type OECTs, P3CBBL-based *n*-type OECTs and P3CPT-based *p*-type OECTs (adapted from [46] with permission from John Wiley and Sons).

As another transistor concept, OECT is emerging where the drain current (I_D_) is controlled depending on the ions electrochemically formed by a liquid electrolyte. In 2020, Zabihipour et al. reported OECT-based circuits using screen-printing [44]. The OECTs were fabricated using poly (3,4-ethylenedioxythiophene) and poly (styrensulfonate) (PEDOT:PSS) with a plastic substrate of polyethylene terephthalate (Figure 2a). This process achieved an excellent process yield as high as 99.7%, while 400 A/A of a switching current ratio was obtained. Furthermore, 760 devices in a single substrate were manufactured using Ag and PEDOT:PSS as a metal line and a gate electrode, respectively, showing the feasibility of implementing OECT-based integrated circuits. As another example of OECT-based circuits, Jo et al. presented gelatin-hydrogel-based OECTs and their applications in various logic gates, including inverters, NAND, and NOR (Figure 2b–d) [45]. The mentioned logic circuits and pH-dependent logic behaviors were demonstrated using the proposed gelatin-based OECTs. The maximum output voltage of the OECT-based NAND gate decreased to 1.11 V, 1.10 V, and 0.5 V, respectively, when increasing from pH = 1.13 to 5.23 and 13.43. Overall, OECTs offer biocompatible electronic applications as electrolytes react with pH and ion concentration.

The two OECT-based digital circuits introduced earlier dealt only with *p*-type OECT. For OECT to be extended to the circuit level and applied to practical applications, a complementary circuit with an *n*-type OECT is required. Although *n*-type OECTs have been limited by the absence of a candidate group with stability in air and aqueous electrolyte media, the development of a complementary circuit consisting only of OECTs began as the *n*-type OECT using a naphthalene-diimide (NDI)-based polymer was first reported. Sun et al. developed an *n*-type OECT by applying poly (benzimidazobenzophenanthroline) (BBL), a ladder-type conducting polymer, as a channel material for the OECT (Figure 2e) [46]. The proposed BBL-based *n*-type OECT, which had a high transconductance of 9.7 mS, showed excellent stability in aqueous media. They further constructed a complementary inverter using the BBL-based *n*-type OECT and the conjugated polyelectrolyte poly- (3-carboxy-pentyl-thiphene) (P3CPT)-based *p*-type OECT (Figure 2f). The OECT-based complementary inverter was successfully demonstrated, which was the first complementary inverter composed of only OECTs for both *p*-type and *n*-type.

### 2.3. Organic-Based Thin-Film Digital Circuits

OTFTs can be fabricated by deposition and solution processes, such as spin coating [47,48] and printing techniques [49,50]. The aforementioned deposition and solution processes for fabricating OTFTs also have potential as components for flexible electronics using substrates with inherently low thermal stability due to their relatively low process temperatures. Accordingly, many researchers have continued to implementing circuit levels using organic semiconductors as an essential step toward OTFT-based practical devices.

**Figure 3:**
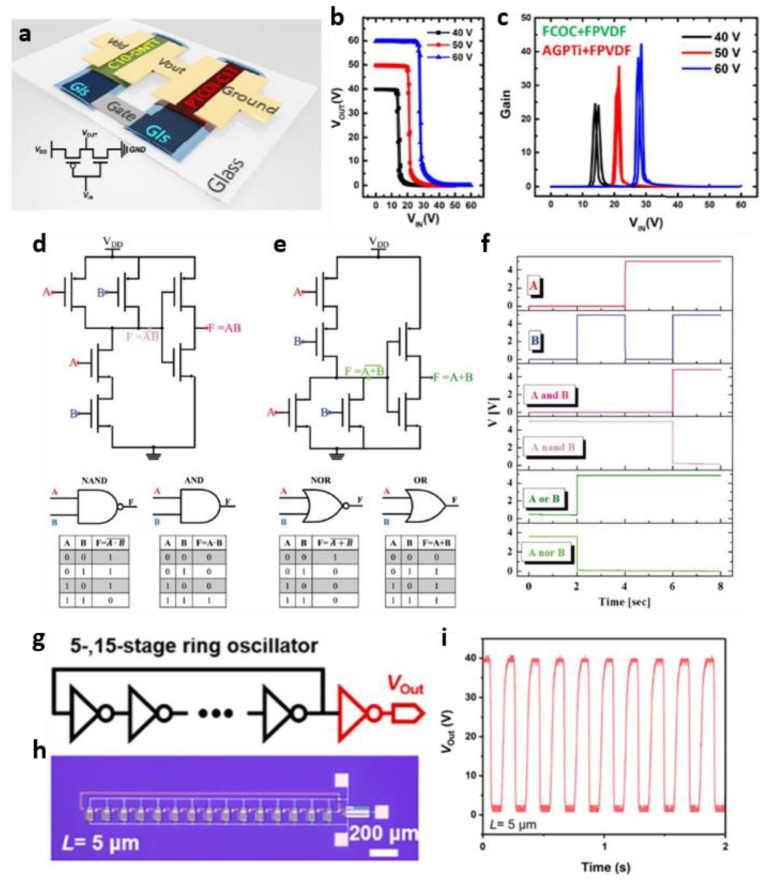
Organic-based digital thin-film circuits. (**a**) Schematic diagram of OTFT-based complementary inverter with a gate dielectric formed by selective printing and its corresponding (**b**) voltage transfer characteristics and (**c**) gain (adapted from [51] with permission from the American Chemical Society). (**d**) Circuit schematic, symbol, and truth table of (**d**) NAND, AND and (**e**) NOR, OR composed of N1400-based *n*-type OTFT and TIPS-Pentacene-based *p*-type OTFT. (**f**) Two different input voltages (A, B) and corresponding output voltages of AND, NAND, OR, and NOR circuits (adapted from [54] with permission from John Wiley and Sons). (**g**) Schematic symbol, (**h**) optical microscopy image, and (**i**) output characteristics of 15-stage organic ring oscillator (adapted from [55] with permission from the American Chemical Society).

First, Tang et al. demonstrated the patterning of various gate dielectrics on gate electrodes using electrohydrodynamic (EHD) printing [51]. EHD printing is commonly used as a non-contact coating method [52,53], but the authors used a direct-contact coating method to cover the gate electrode completely. Four printing gate dielectrics were used: poly (vinylidene fluoro-co-hexafluoropropylene) (PVDF-HFP), fluorophenyl azide (FPA)-3F-based cross-linked PVDF-HFP (FPVDF-HFP), cyclic olefin copolymer (COC) with FPA-3F (FCOC), and an organic–inorganic hybrid material named AGPTi. In addition, six gate dielectrics, including a bilayer dielectric, were fabricated and characterized by applying them to (2,9-didecyl-dinaphtho-[2,3-b:2′,3′-f]-thieno-[3,2-b]-thiophene (C10-DNTT) and N′-ditridecylperylene-3′,4,9,10- tetracarboxylic diimide (PTCDI-C13) OTFTs. Finally, they implemented a digital logic circuit. The *p*-type and *n*-type OTFTs had a selectively patterned bilayer gate dielectric, which is the first demonstration result. The gate dielectric can be patterned selectively by targeting each OTFT, meaning that organic semiconductors with different properties can be optimized. Figure 3a shows a schematic diagram of the fabricated OTFT-based complementary logic inverter. Figure 3b,c show the application of a bilayer of a combination of FOC and RPVDF as a gate dielectric of C10-DNTT OTFT and a combination of AGPTi and FPVDF as a gate dielectric of PTCDI-C13 OTFT. The implemented logic inverter performed a full-swing function from the ground to *V*_DD_. Casula et al. implemented an OTFT-based flexible logic gate on a paper substrate using organic materials for the gate dielectric and semiconductor layers and the gate and source/drain electrodes [54]. Both *p*-type and *n*-type OTFTs constituting the logic gate showed that the saturation mobility did not deteriorate significantly and was maintained stably, even after 200 bending cycles with a bending radius of 0.75 cm and a bending radius of at least 0.5 cm. Based on this stable OTFT, they demonstrated basic logic gates, such as NAND, NOR, AND, and OR gates. Figure 3d shows the circuit schematic extended to the AND gate by inverting the NAND gate output by configuring an inverter in the NAND gate. Similarly, they extended from the NOR gate to the OR gate (Figure 3e). The constructed logic gates correctly outputted the operation results that correspond to the truth table of each logic gate in Figure 3f for two input voltages (A and B). Chen et al. demonstrated that it is possible to pattern organic semiconductors with a high yield by developing an anti-solution penetration photolithography process [55]. This was achieved using highly cross-linked semiconductor films to prevent the dissolution of the low-ordered region, and through delicate patterning they implemented a 15-state ring oscillator (Figure 3g,h). This ring oscillator contains the most transistors among all photolithography organic electronics based on the reported photo-cross-linkable organic semiconductors. Figure 3i shows that the output characteristics of the fabricated 15-state ring oscillator have an oscillation frequency of approximately 6 Hz. This is a slow speed compared to the previously reported organic ring oscillator. On the other hand, such all-photolithography integrated organic circuits can be developed at a faster frequency if the organic-based semiconductor and gate dielectric are optimized and scaled down.

### 2.4. Metal-Oxide-Based Thin-Film Digital Circuits

**Figure 4:**
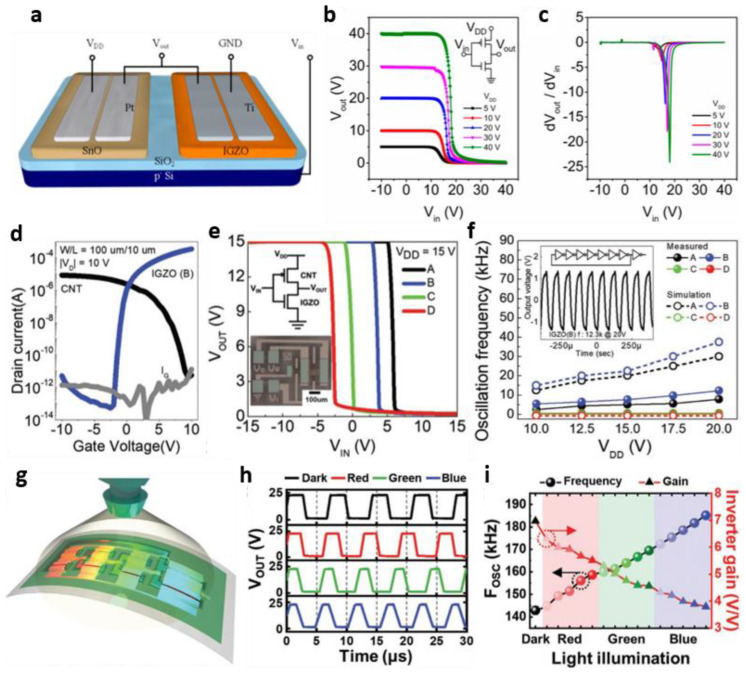
Metal-oxide-based digital thin-film circuits. (**a**) Structural illustration, (**b**) voltage transfer characteristics, and (**c**) gain of inverter composed of SnO and IGZO TFTs (adapted from [68] with permission from MDPI). (**d**) Transfer curves for optimal a-IGZO and SWCNT TFTs. € The voltage transfer characteristics of the CMOS inverter according to the deep ultraviolet irradiation time on IGZO TFT. (**f**) Simulated and measured oscillation frequencies as a function of *V*_DD_, and output characteristics of a CMOS seven-stage ring oscillator, when *V*_DD_ = 20 V (adapted from [71] with permission from John Wiley and Sons). (**g**) Illustration in which light is irradiated to a three-stage ring oscillator based on a-IGZO and SWNT TFTs. (**h**) Output waveform, (**i**) oscillation frequency and gain of a three-stage ring oscillator according to the wavelength of light irradiated, such as red, green, and blue (adapted from [72] with permission from John Wiley and Sons).

High-performance TFTs have been developed mainly for *n*-type TFTs such as indium–gallium zinc oxide (IGZO) [56,57], indium oxide (In_2_O_3_) [58,59], and zinc oxide (ZnO) [60,61], owing to the intrinsic properties of metal-oxide-based semiconductors. Nevertheless, many researchers have tried implementing *p*-type metal oxide TFTs to realize metal-oxide-based complementary digital circuits. As a result, *p*-type metal oxide semiconductors, such as tin monoxide (SnO) [62,63], copper (ii) oxide (CuO) [64,65], and nickel oxide (NiO) [66,67], have been developed, even though they are poor compared to *n*-type metal oxide semiconductors. Along with developing metal oxide TFTs with *p*-type characteristics, attempts were made to implement complementary circuits using only metal-oxide-based TFTs. In 2017, Zhang et al. reported a metal oxide-based complementary inverter (Figure 4a) [68]. *p*-type and *n*-type TFTs were fabricated by depositing SnO and IGZO using RF sputtering and patterning with shadow masks. The proposed inverter showed full-swing voltage transfer characteristics with respect to all five *V*_DD_ (= 5, 10, 20, 30, and 40 V) (Figure 4b). The DC gain of the inverter composed of SnO and IGZO was 24 V/V when the *V*_DD_ was 40 V, which showed better characteristics than other inverters composed only of metal oxide semiconductors (Figure 4c). Therefore, Zhang et al. suggested that the combination of *p*-type SnO and *n*-type IGZO is promising in fully oxide-based electronics.

As briefly mentioned above, metal-oxide-based semiconductors inherently have poor *p*-type characteristics. A combination of organics and CNTs, where *p*-type is relatively dominant, has been frequently selected to compensate for these shortcomings and advance the metal oxide TFT to the circuit level [69,70]. Kim et al. implemented up to the circuit level by combining *n*-type IGZO TFT with *p*-type SWCNT TFT [71]. Both IGZO and SWCNT layers were fabricated by a solution-based spin coating method. In the case of IGZO, a doping process through deep ultraviolet (DUV) irradiation was also applied. At this time, the samples annealed for 90, 120, 150, and 180 min were classified as A, B, C, and D, respectively. Figure 4d shows the transfer curves of the fabricated IGZO TFT and SWCNT TFT, and both devices had a sufficient on/off drain current ratio of about 10^6^ A/A or more. In particular, in the case of IGZO TFT, as the DUV irradiation time increased, the carrier concentration in the channel increased, leading to formation at a position where the *V*_th_ of the transfer curve was more negative. As a result, the voltage transfer characteristics of the logic inverter composed of IGZO TFT and SWCNT TFT were also negatively shifted (Figure 4e). Nevertheless, regardless of the DUV doping time of IGZO, the logic inverters in all cases had a DC gain of 22 V/V or more and a noise margin of 90% or more. They further demonstrated a seven-stage ring oscillator from a logic inverter and showed an oscillation frequency of approximately 12.3 kHz when *V*_DD_ was 20 V under optimal doping conditions (Figure 4f).

As another example, Jeong et al. also implemented digital circuits, such as logic inverters, NAND, NOR, and ring oscillators, using a combination of IGZO and SWCNT TFTs [72]. They demonstrated light-to-frequency conversion circuits (LFCs) in which the light modulated the oscillation frequency. To implement the LFC that responds to visible light, they intentionally formed many oxygen vacancies to improve the photoresponse characteristics of IGZO TFT. Therefore, IGZO was heat treated at a low temperature. As a result, IGZO, with a large energy bandgap of approximately 3.2 eV, could respond to visible light. They used red (656 nm), green (530 nm), and blue (455 nm) light as visible light to modulate the frequency of the ring oscillator. Figure 4g shows the situation in which each light was irradiated to the fabricated three-state ring oscillator, and the output characteristics at that time are shown in Figure 4h. In the dark, the oscillation frequency, 143 kHz, increased to 159 kHz, 170 kHz, and 185 kHz when irradiated with red, green, and blue lights (Figure 4i). At this time, the wavelength of light and the oscillation frequency showed a linear relationship, which means that the calibration process can be omitted when composing the LFC circuitry later, which leads to the system being further simplified.

### 2.5. TMDs-Based Thin-Film Digital Circuits

TMDs are the most emerging material among next-generation semiconductor materials. There are many candidates with their own *p*-type and *n*-type characteristics, unlike organic and metal oxide semiconductors. Therefore, high-performance circuit design is only possible with TMDs. In addition, the optical and electrical properties dependent on the number of layers have the advantage that more functions can be induced in the circuit design. In particular, the two-dimensional characteristics of TMDs are widely applied to devices by combining them with graphene, a material of the same dimension.

**Figure 5:**
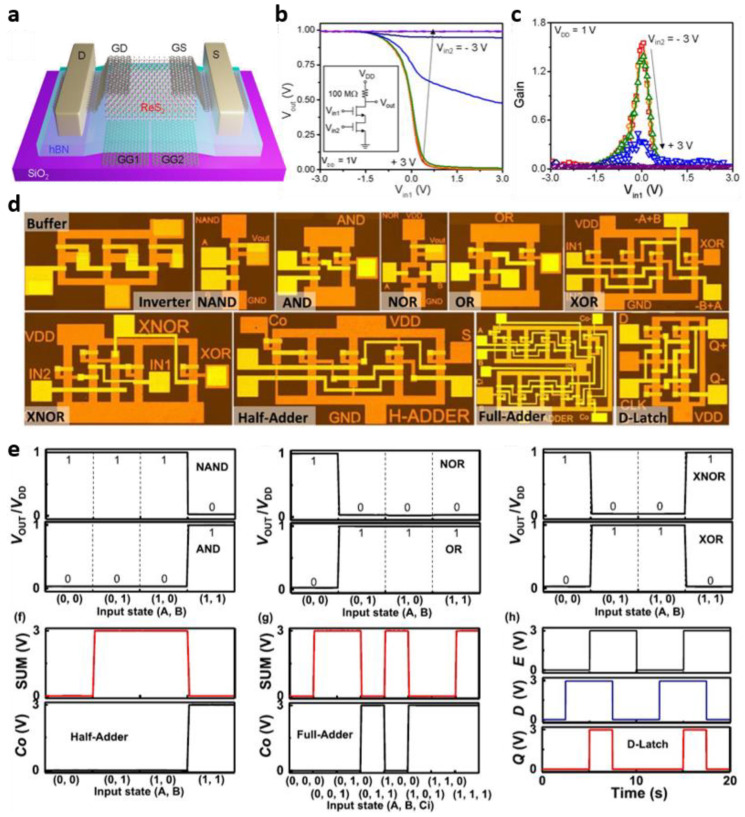
TMD-based digital thin-film circuits. (**a**) Structural schematic diagram of ReS_2_ TFT with graphene split gates. (**b**) Input/output characteristics and (**c**) corresponding gain of a logic circuit consisting of a resistor and a ReS_2_ FET with two graphene split gates (adapted from [73] with permission from Springer Nature). (**d**) Various digital circuits, such as buffers and inverters, based on MoS_2_ TFT and (**e**) input/output characteristics corresponding to each (adapted from [74] with permission from the American Chemical Society).

Kwon et al. implemented a logic NAND gate with one ReS_2_ TFT by introducing graphene split gates to modulate the channel region partially [73]. Figure 5a shows a ReS_2_ TFT with graphene split gates. The two gate electrodes, *V*_in1_ and *V*_in2_, are independently controlled, and if a negative voltage is applied to one of the gates, the entire device is turned off. This characteristic was also confirmed through the logic operation shown in Figure 5b. When *V*_in2_ is −3 V, logic “1” is output regardless of *V*_in1_. On the other hand, when *V*_in2_ was 3 V, inverter characteristics were shown according to *V*_in1_ (Figure 5b). The DC gain was modulated in response to the output characteristics that vary according to the combination of the two gate voltages (Figure 5c). A logic “0” is output only when both gate voltages are 3 V, indicating that one ReS_2_ TFT can act as a NAND gate.

Furthermore, as an example of implementing a more complex digital circuit using MoS_2_ among TMDs, Ma et al. proposed a non-destructive doping method in which the electrical properties are controlled according to the Al thickness when a bilayer structure, such as Al/Au, is adopted as the gate electrode for MoS_2_ synthesized through the CVD process [74]. By applying the optimized doping conditions, they implemented more complex digital logic circuits, such as full-adder and D-latch beyond MoS_2_ TFT-based buffer and inverter (Figure 5d,e). This shows the maturity of CVD technology for 2D semiconductors and, simultaneously, can provide a clue as a solution to the scale-down approach facing the Si-based semiconductor industry, which is currently reaching its limit.

## 3. Analog circuits

### 3.1. CNT-Based Thin-Film Analog Circuits

**Figure 6:**
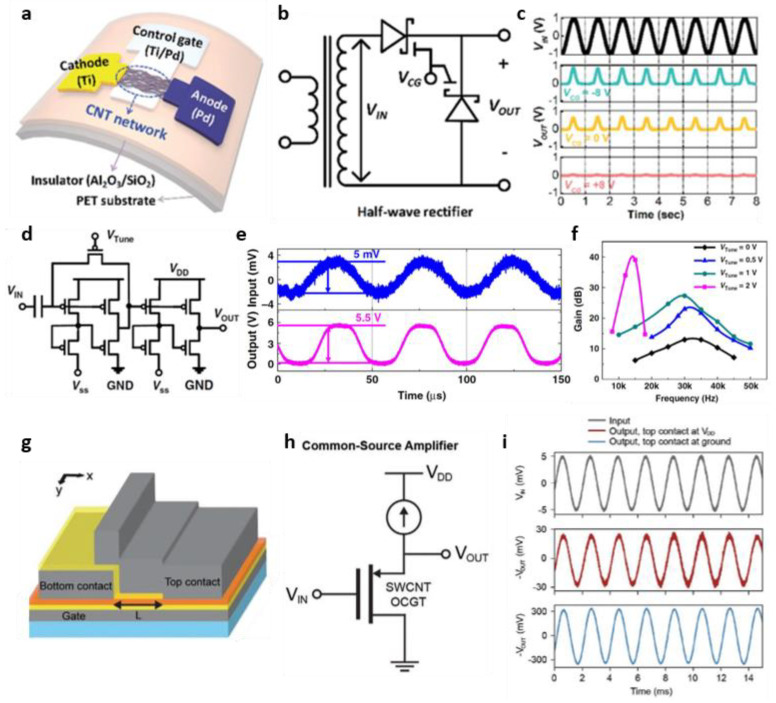
CNT-based thin-film analog circuits. (**a**) Structural illustration of the flexible CNT Schottky diode with a control gate. (**b**) Schematic diagram of a half-wavelength rectifier circuit composed of two CNT Schottky diodes and (**c**) rectification characteristics dependent on the control gate voltage (adapted from [75] with permission from RSC Publishing). (**d**) Circuit diagram of CNT-based amplifier with post-fabrication tunability through voltage of *V*_Tune_ node. (**e**) Output characteristics when an input signal with a peak-to-peak voltage of 5 mV is applied to the amplifier. (**f**) When V_Tune_ is adjusted within 0 V to 2 V, the corresponding gain and bandwidth (adapted from [76] with permission from Springer Nature). (**g**) Structural schematic diagram of CNT-based ohmic-contact-gated transistor. (**h**) Circuit diagram of a common source amplifier using a CNT-based ohmic-contact-gated transistor and (**i**) its input/output characteristics according to the position of the voltage-applying electrode (adapted from [77] with permission from John Wiley and Sons).

This section reports an example of implementing an analog circuit using CNTs. Lee et al. fabricated a gate-tunable Schottky diode with a semiconducting CNT-based asymmetric contact structure obtained using the density–gradient ultracentrifugation method (Figure 6a) [75]. In addition, they fabricated a gate-tunable Schottky diode on a flexible substrate (PET) and showed stable characteristics without deterioration in electrical characteristics even after 5000 bending cycles. Using a gate-tunable Schottky diode with such stable characteristics, digital circuits, such as OR and AND gates, and analog circuits, such as half-wave rectifiers, were demonstrated. Figure 6b presents the fabricated half-wave rectifier. The loss of the AC input voltage was almost eliminated as the gate voltage increased in the negative direction (Figure 6c). Therefore, the rectifier efficiency of the demonstrated half-wave rectifier was 39.3%, which is close to the ideal value of 40.6%.

All circuits should be basically kept for each purpose, and even if it is the same circuit, the usefulness of the circuit can be further increased if the function of the circuit can be adjusted according to the situation after being manufactured. Lei et al. extracted semiconducting CNTs with a high yield based on the polymer-sorting technique; only one CNT out of 34,590 CNTs was a metallic tube, and the corresponding purity reached 99.997% [76]. Using high-purity semiconducting CNTs, they first implemented digital logic gates, such as inverters, NAND, and XOR gates. Furthermore, they implemented more complex digital circuits, such as D flip-flop and shift resistors, as well as CNT TFT-based amplifiers (Figure 6d,e). Even after the proposed amplifier was fabricated, it was possible to control the parameters, such as voltage gain and bandwidth, through the voltage of the *V*_TUNE_ node (Figure 6f).

Rojas et al. fabricated a CNT-based TFT with a new structure called an ohmic contact gated transistor (OCGT), as shown in Figure 6g [77]. Unlike the existing source-gated transistor, which requires a channel length longer than depletion, OCGT can implement a short channel of 300 nm or less and enables current saturation despite the short channel. They implemented and evaluated the OCGT-based common source amplifier to prove the applicability of the proposed CNT-based OCGT as a practical device (Figure 6h,i). The common source amplifier was measured in two cases: the case where the top contact was ground and the case where the bottom contact was ground. As a result, the amplified output voltage was obtained in both cases. For *V*_in_, with an amplitude of 5 mV, the voltage gain was 5 *V*/*V* when the bottom contact was ground and 68 *V*/*V* when the top contact was ground, respectively. The difference in voltage gain between the two cases appears to be due to the difference in transconductance at each mode.

### 3.2. OECT-Based Thin-Film Analog Circuits

**Figure 7:**
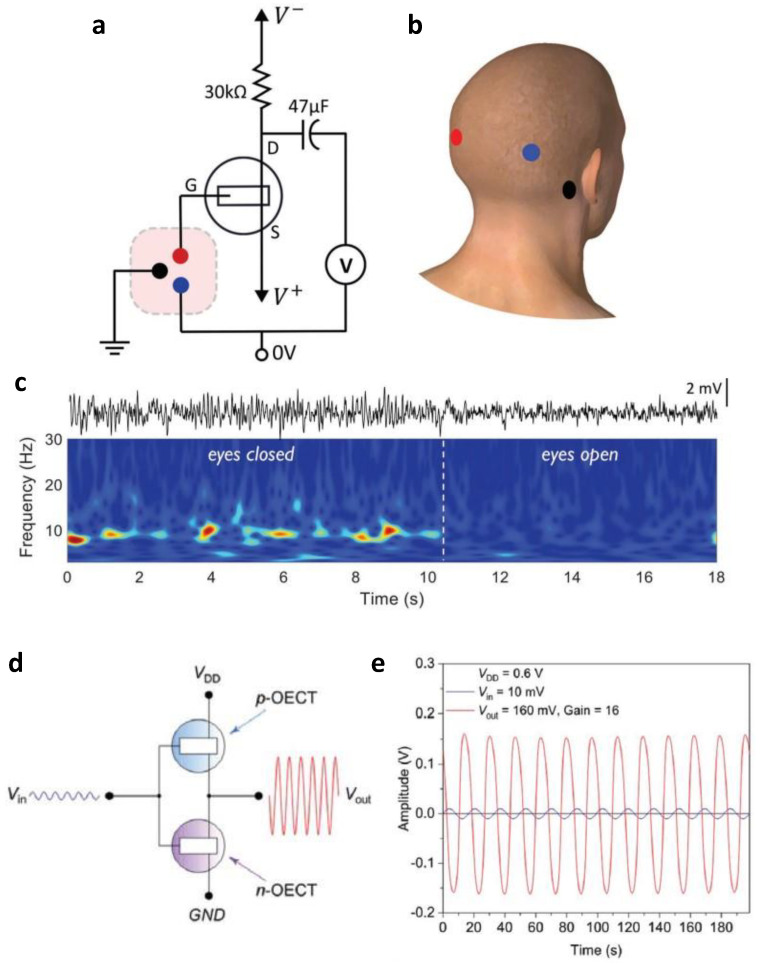
OECT-based thin-film analog circuits. (**a**) A sub-threshold OECT-based voltage amplifier for EEG detection, and (**b**) the detailed location on the scalp of a human to which each electrode is attached. (**c**) Comparison of the observation of the alpha rhythm response, normally activated when the eyes are closed, by converting the EEG signals when the eyes are closed and when they are open into time-frequency plots (adapted from [78] with permission from John Wiley and Sons). (**d**) Circuit diagram of OECT-based complementary amplifier and (**e**) corresponding input/output characteristic (adapted from [79] with permission from John Wiley and Sons).

Unlike general transistors, OECT, which has an electrolyte as a gate dielectric, is stable in an aqueous environment and is suitable for sensing biosignals. For this reason, OECT has been mainly studied for amplification applications to amplify biological signals. Venkatraman et al. analyzed in depth the operation region of OECT using p(g2T-TT), a *p*-type OECT material [78]. They emphasized low power consumption rather than high gain in OECT-based amplifiers. They suggested that operation in the sub-threshold region with high transconductance efficiency is required to achieve this. In this regard, the depletion mode, PEDOT:PSS-based OECT, requires a relatively high positive voltage to reach the subthreshold region. On the other hand, this bias condition was limited in aqueous conditions due to the hydrolysis issue. The p(g2T-TT)-based OECT operates in the enhancement mode and can easily reach the subthreshold region. Finally, the human electroencephalogram (EEG) signal was sensed using an OECT-based amplifier (Figure 7a,b). Converting the measured EEG voltage into a time-frequency plot confirmed that the alpha rhythm that appears when the eyes are closed, corresponding to approximately 10 Hz, is observed only when the person’s eyes are open (Figure 7c). Furthermore, the possibility of utilizing the subthreshold region of OECT was assessed by demonstrating the electrocardiography (ECG) measurements.

Unipolar TFT-based circuits have many limitations because of power consumption due to the static current. A method of forming an operating point in the subthreshold region described above or a complementary circuit is required to solve this problem. Therefore, *p*-type OECT and *n*-type OECT should be able to form a circuit together. Yang et al. implemented an OECT-based complementary circuit with a side gate structure rather than a top gate method [79]. Polythiophene functionalized with tetraethylene glycol side chains (P(g_4_2T-T)) and polycationic polyquaternium-10 (PQ-10) hydrogels were used as channels and electrolytes for *p*-type OECT, and BBL and polyanionic poly (sodium-4-styrene sulfonate) (PSSNa) hydrogels were used for *n*-type OECT. To match the current level between *p*-type OECT and *n*-type OECT, they optimized the thickness of each type of active layer. A complementary inverter-type OECT amplifier was manufactured based on the balanced drain current level (Figure 7d). Complementary inverter-based amplifiers can achieve high gain characteristics, but it is challenging to set an accurate operating point. To characterize the manufactured OECT-based amplifier, the output signal amplitude was 160 mV when an AC signal with an offset voltage of 0.28 V and an amplitude of 10 mV was applied. Therefore, in this case, the voltage gain obtained was 16 *V*/*V*, as shown in Figure 7e. A 33 *V*/*V* voltage gain was observed when the amplitude was 200 μV. As such, the OECT-based circuit has great potential in wireless healthcare devices that can be monitored continuously because of its low power and low voltage operation advantages.

### 3.3. Organic-Based Thin-Film Analog Circuits

**Figure 8:**
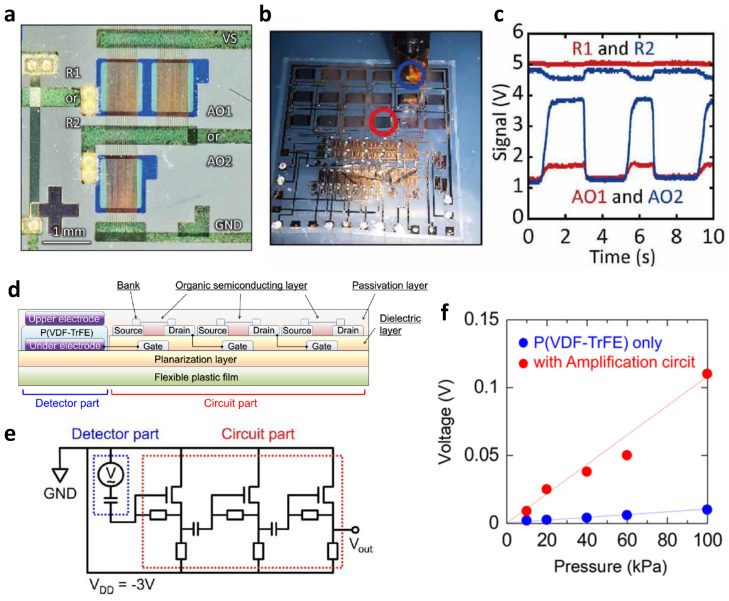
Organic-based thin-film analog circuits. (**a**) An optical microscope image of a common source amplifier, one of the elements constituting an organic-based active magnetosensory matrix system. (**b**) An image of a magnet approaching one of the GMR sensors away from each other and (**c**) the signal before and after amplification of the two GMR sensors at this time (adapted from [80] with permission from AAAS publications). (**d**) Structural schematic and (**e**) circuit diagram of a highly pressure-sensitive printed sensor consisting of a pressure-sensing part based on P(VDF-TrFE) and a circuit amplifying the sensed signal. (**f**) Comparison of the final generated voltage with and without the amplifier circuit when the same amount of pressure change is sensed in the P(VDF-TrFE) layer (adapted from [81] with permission from American Chemical Society).

In the case of organic semiconductors, a *p*-type OTFT with excellent performance and atmospheric stability has been developed because it is vulnerable to electron trapping by atmospheric elements, such as H_2_O or O_2_, but an *n*-type OTFT is still insufficient. Therefore, most organic-based complex circuits have only consisted of *p*-type organic semiconductors. Kondo et al. demonstrated an organic-based active magnetosensory matrix (MSM) system [80]. They configured the bootstrap shift registers, signal amplifiers, and current mirrors, which are necessary elements for the operation of the MSM system, with DNTT TFT, a *p*-type organic semiconductor. Because of independently driving each OTFT-based component, it operated normally, and the MSM system also detected the magnetic field. For most sensor devices, including the MSM system, it is essential to amplify the sensed minute signal to a level that can be processed. Therefore, an amplifier was also used in this MSM system (Figure 8a). The role of the amplifier in the proposed MSM system is shown more clearly in Figure 8b,c. Figure 8b shows the magnet approaching the giant magnetoresistance (GMR) sensor in the blue circle, and Figure 8c compares voltage signals at two different positions. Before amplifying the detected signal, the difference between the signals for different GMR sensors (R1, R2) was insignificant. On the other hand, after each signal was amplified, it was possible to clearly distinguish which GMR sensor the magnet was close to through the difference between the signal of the GMR sensor that was close to the magnet (AO2) and the signal of the GMR sensor that was far away from the magnet (AO1).

As an example of another application using a *p*-type OTFT-based amplifier, Sekine et al. introduced an OTFT-based amplifier to amplify the signal sensed from a pressure sensor built based on PEDOT:PSS and poly(vinylidene fluoride-co-trifluoroethylene) (P(VDF-TrFE)) [81]. They used dithieno [2,3-d;2′,3′-d′]benzo[1,2-b;4,5-b′]dithiophene (DTBDT-C6) as a channel layer for OTFT by printing. Figure 8d,e shows the overall sensor system structure and circuit schematic. In this structure, an electrode of the part that detects the pressure is connected to the input terminal of the amplifier. The output voltage of the manufactured pressure sensor, according to the presence of an amplifier, was compared to verify the effect of the amplifier (Figure 8f). In both cases, a linear change in output voltage was observed with pressure regardless of the amplifier. On the other hand, when an amplifier was added, a more considerable output voltage change was obtained for the same change in pressure. Based on the sensitivity of the pressure sensor improved through the amplifier, they finally measured the human electrocardiogram (ECG) signal and successfully extracted the pulse wave velocity (PWV).

### 3.4. Metal-Oxide-Based Thin-Film Analog Circuits

**Figure 9:**
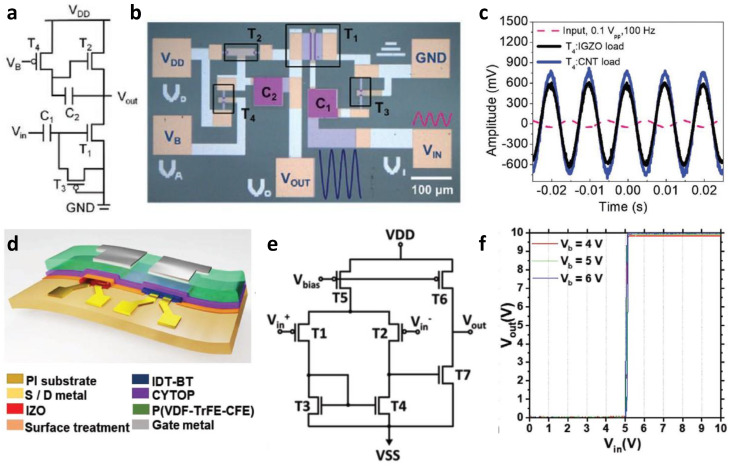
Metal-oxide-based thin-film analog circuits. (**a**) Circuit schematic and (**b**) optical microscopy image of a CMOS amplifier composed of IGZO and CNT TFTs. (**c**) Comparison of the input/output characteristics according to whether the load transistor was an IGZO TFT or a CNT TFT in a CMOS amplifier with the same configuration (adapted from [82] with permission from John Wiley and Sons). (**d**) Complementary circuit schematic diagram composed of IZO and IDT-BT. (**e**) The circuit diagram of a two-stage amplifier composed of IZO and IDT-BT and (**f**) the corresponding voltage transfer characteristics. (adapted from [83] with permission from John Wiley and Sons).

As mentioned earlier in the digital circuit section, *n*-type metal oxide TFTs have been developed, whereas the development of *p*-type metal oxide TFTs has been limited. Although there are CuO-, SnO-, and NiO-based TFTs as *p*-type metal oxide TFTs, they are limited in implementation in a complementary structure due to inferior electrical properties compared to *n*-type TFTs. To overcome this problem, many researchers have implemented complementary circuits to overcome this problem by combining CNT and organic-based TFTs with predominant *p*-type characteristics with *n*-type metal oxides. Kim et al. implemented a complementary amplifier using *n*-type IGZO TFT and *p*-type CNT TFT [82]. Figure 9a,b shows the circuit diagram and an optical microscopic image of the fabricated amplifier. They found that the gain and transient response characteristics of the implemented amplifier were closely related to the output impedance through simulation. They reported that they were proportional to the resistance of the load transistor T4. Therefore, they adapted a CNT TFT with higher resistance derived from a lower mobility as T4 to achieve high gain. As a result, an output signal with a larger amplitude was obtained when the CNT TFT was positioned at T4 compared to when the IGZO TFT was set to T4 (Figure 9c).

Another example of implementing an analog circuit using an *n*-type metal oxide TFT and a *p*-type TFT based on a material other than metal oxide is as follows. Kang et al. fabricated an OTFT and hybrid analog circuit based on IDT-BT, a *p*-type organic semiconductor, to verify the improved performance of *n*-type IZO TFT through an ultraviolet light–negative oxygen ion surface passivation treatment (UVNOI treatment) (Figure 9d). Figure 9e shows the circuit diagram of the differential amplifier composed of IZO TFTs and IDT-BT TFTs. The current mirror (T3, T4) and driving transistor (T7) of the manufactured differential amplifier were all composed of IZO TFT. Before the UVNOI treatment of the IZO TFT, the output of the amplifier could not fully swing from the ground to *V*_DD_. On the other hand, full swing from ground to 9.93 V and a gain of approximately 100 *V*/*V* were obtained from very sharp switching characteristics from the improved performance of IZO TFT through UVNOI treatment, as shown in Figure 9f.

### 3.5. TMD-Based Thin-Film Analog Circuits

**Figure 10:**
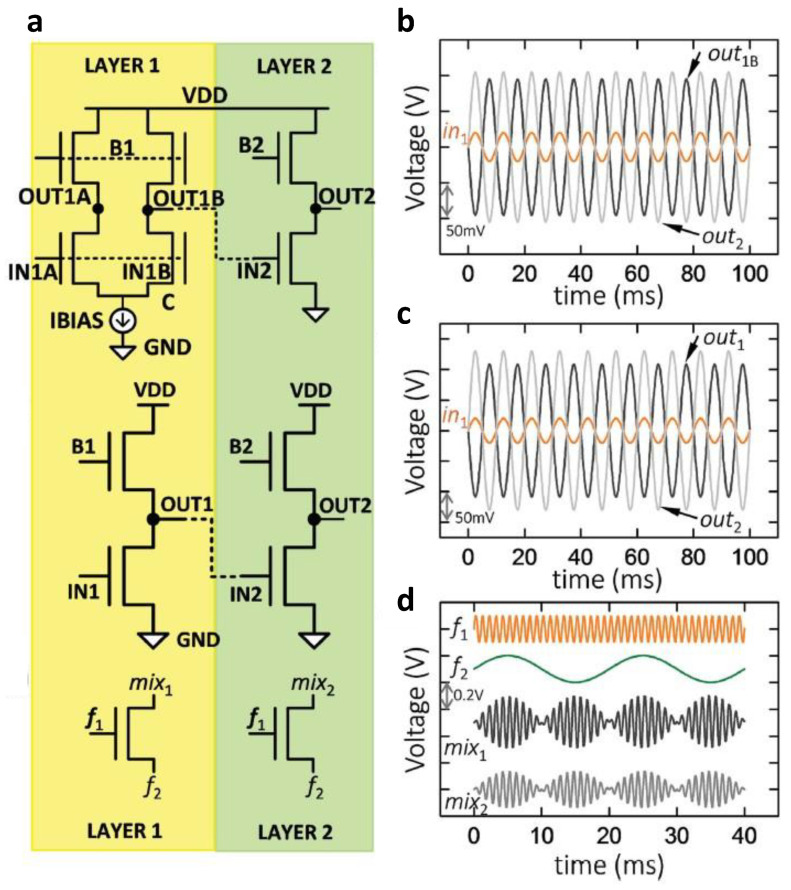
TMD-based thin-film analog circuits. (**a**) Circuit schematics of the differential amplifier, common source amplifier, and signal mixer composed of MoS_2_ and WSe_2_ TFTs, and (**b**–**d**) input/output characteristics corresponding to each circuit (adapted from [84] with permission from John Wiley and Sons).

Unlike CNT, organic, and metal oxide TFTs, which have implemented digital and analog circuits, circuits using 2D TMDs are mainly concentrated on digital circuits. In 2016, Sachid et al. implemented a complementary circuit by vertically stacking *n*-type MoS_2_ and *p*-type WSe_2_ TFTs [84]. Digital and analog circuits were also implemented on a vertically integrated structure capable of improving the integration density, providing more functionality in a limited area. They implemented an inverter, NAND, and NOR for digital circuits, and differential amplifiers, common source amplifiers, and signal mixers were implemented for analog circuits (Figure 10a). The input/output characteristics of each analog circuit are shown in Figure 10b-d. This result was significant in demonstrating digital and analog circuits with TMD-based TFTs, one of the 2D materials. On the other hand, manufacturing methods, such as mechanical exfoliation, had difficulties extending to more complex analog circuits.

Research on improving the uniformity and synthesizing large areas of TMDs has been continuously conducted, and a MoS_2_-based operational amplifier (Op-Amp) was implemented in 2020. Polyushkin et al. implemented a MoS_2_-based Op-Amp grown using CVD [85]. Op-Amp is a building block that plays a key role in analog circuits, and the successful fabrication of Op-Amp means that various analog functions can be expanded. They demonstrated various arithmetic circuits, such as inverting amplifiers, integrators, and logarithmic amplifiers, by combining passive elements, such as resistors and capacitors, with the implemented Op-Amp and configuring feedback. This showed the potential for developing analog circuits based on 2D materials, and it is expected to approach practical applications using 2D TMDs through continued research.

## 4. Summary

In this review, we reported on the progress from the unit device level to the circuit level using TFTs based on CNTs, OECTs, organics, metal oxides, and TMDs. TFTs, which are fabricated by a bottom-up method rather than a top-down method, have weaknesses in charge mobility and scaling compared to conventional MOSFETs, but have advantages in terms of large area and low-cost process. In particular, TFT has high substrate compatibility with various substrates such as transparent glass, flexible plastic, and paper. Therefore, TFTs have great potential as a device component in healthcare monitoring systems and biosensors that require flexible and wearable characteristics. Furthermore, since TFTs can be fabricated by combining various materials such as CNTs, OECTs, organic materials, metal oxides, and TMDs, target mechanical and electrical properties can be achieved by selectively choosing materials. Recently, in addition to material combinations in the TFT itself, attempts have been made to integrate different devices, such as LEDs, photodetectors, etc., with the TFTs [86,87,88].

As such, TFT has many advantages over MOSFET, but some issues must be overcome to advance to useful and practical applications. (i) First, high operational stability at the unit device level must be secured. (ii) Secondly, it is necessary to minimize device-to-device variation and batch-to-batch variation on a large-area substrate and process optimization for high yield. The uniformity and process yield of TFTs are critical to the operation of a circuit because if any of the many TFTs that make up the circuit fail, the circuit cannot function properly even if all other TFTs work without malfunction. (iii) Third, the metal routing method for interconnection between different TFTs should also be studied. This is because the conventional method for forming via-holes through laser drilling and etching may cause deterioration of the channel layer, such as organic semiconductors, due to high thermal, mechanical, and chemical damage that occurs during the process. Recently, to solve this issue, a metal routing method using an insulator that can be patterned without a via-hole was reported [89,90]. As such, there is a need for continuous research on a technology that can stably achieve interconnection between metals without damaging the lower layers. Undoubtedly, as mentioned, there are several challenges to overcome before approaching complex systems via TFT-based circuitry. Nevertheless, even at this moment, many researchers are taking steps towards advanced TFT-based applications, and the day will come when their ceaseless efforts will bear fruit in the not-so-distant future.
